# Local Laboratory Testing of Germline BRCA Mutations vs. Myriad: A Single-Institution Experience in Korea

**DOI:** 10.3390/diagnostics11020370

**Published:** 2021-02-22

**Authors:** Joohyun Hong, Jiyun Lee, Minsuk Kwon, Ji-Yeon Kim, Jong-Won Kim, Jin Seok Ahn, Young-Hyuck Im, Yeon Hee Park

**Affiliations:** 1 Division of Hematology-Oncology, Department of Medicine, Samsung Medical Center, Sungkyunkwan University School of Medicine, Seoul 06351, Korea; hhkeith0902@gmail.com (J.H.); jiyun31.lee@samsung.com (J.L.); minsuk.dr.kwon@samsung.com (M.K.); jyeon25.kim@samsung.com (J.-Y.K.); jinseok.ahn@samsung.com (J.S.A.); imyh00@skku.edu (Y.-H.I.); 2 Department of Laboratory Medicine, Samsung Medical Center, Sungkyunkwan University School of Medicine, Seoul 06351, Korea; culture.jkim@gmail.com

**Keywords:** breast carcinoma, genes, mutation

## Abstract

Genetic diagnosis for human epidermal growth factor receptor 2-negative metastatic breast cancer patients with the germline *BRCA* (gBRCA) mutation has been emphasized since the development of polyadenosine diphosphate-ribose polymerase inhibitors. Myriad Genetics, Inc.’s (Salt Lake City, UT, USA) companion diagnostics service is almost exclusively used for genetic testing. The aim of this study was to compare the results of germline *BRCA* mutation tests returned by a local laboratory and those performed by Myriad. Between April 2014 and February 2018, 31 patients with gBRCA 1/2 mutation test results from both Samsung Medical Center (Seoul, Korea) and Myriad were enrolled. “Discordant: Opposite classification” was observed for only one among 27 (3.7%). This discrepancy was due to the detection of a deleterious large genomic rearrangement of *BRCA 1* by Myriad. Samsung Medical Center performed multiple ligation-dependent probe amplifications (MLPA) to detect large genomic rearrangements only in high-risk patients. This one case was not suspected as high risk and MLPA was not performed. The concordant rate was 74.1% for all 27 patients. “Discordant: Laboratory’s uncertain classification” was found in 22.2% of the sample (six patients). All discrepancies were generated during interpretation of *BRCA 2* gene sequencing. Further studies and standardization of genetic testing for *BRCA 1/2* genes are required.

## 1. Introduction

Breast cancer is a leading cause of cancer and cancer-related death in women worldwide [1]. Breast cancer consists of various types of cancer. Although most breast cancer is sporadic, hereditary breast cancer comprises up to 10% of cases [2]. *BRCA 1/2* genes are tumor suppressor genes that contribute to DNA repair and protect DNA from damage [3]. Mutated *BRCA 1/2* genes are known to cause breast cancer. Carriers of germline *BRCA 1* and germline *BRCA 2* mutations have 65% and 45% chances of developing breast cancer by age 70, respectively [4]. Due to the number of breast cancer patients with germline *BRCA* (gBRCA) mutations, not only genetic risk assessment and counseling, but also specific treatments for breast cancer patients with gBRCA mutations, are increasingly emphasized [5].

Several new drugs have been invented through progress in oncogenomics. Olaparib and talazoparib, polyadenosine diphosphate-ribose polymerase (PARP) inhibitors, are associated with significant improvements of progression-free survival for patients with gBRCA mutation human epidermal growth factor receptor 2 (HER2)-negative metastatic breast cancer [6,7]. The PARP inhibitor successfully affects gBRCA mutation HER2-negative metastatic breast cancer by so-called synthetic lethality [8]. Such innovative development of PARP inhibitors has naturally increased the importance of companion diagnostics because gBRCA mutation positivity is necessary to administer these novel drugs.

The gBRCA mutation test by Myriad Genetics, Inc. (Salt Lake City, UT, USA) is almost exclusively used as the sole accepted diagnostic test. A companion diagnostic (CDx) from Myriad, BRACAnalysis^®^ CDx, characterizes the entire *BRCA 1* and *BRCA 2* genes. It utilizes the polymerase chain reaction (PCR) and Sanger sequencing, and the concordant rate is 100% when compared to next-generation sequencing [9]. In addition, large genomic rearrangements, such as deletions and duplications in *BRCA 1* and *BRCA 2* are detected using multiplex PCR. However, compared to gold standard microarray-based tests, the overall percentage agreement was 94% [9]. This suggests the apparent limitation of false negative results for the BRACAnalysis^®^ CDx Large Rearrangement test. Variants discovered by this test are classified into one of five categories: deleterious mutation, suspected deleterious mutation, variant of uncertain significance, favor polymorphism and polymorphism. The Food and Drug Administration of the United States approved BRACAnalysis^®^ CDx including Myriad’s classification process, considering that novel mutations are still being identified and classified. It is possible that deleterious mutations will change and be reclassified over time [9]. However, there has been criticism because Myriad held a patent for isolated *BRCA 1/2* genes and utilized genomic data exclusively for their own profit [10]. After years of lawsuits, Myriad lost their patent on isolated *BRCA 1/2* genes, and many other corporations were able to develop their own CDx for gBRCA mutations after 2013. However, the gBRCA mutation test developed by Myriad is still the most popularly used due to its high accuracy, the result of development using data accumulated over more than a decade [11]. Not only sequencing of genes, but also interpretations of sequencing results, are becoming more important because of conflicting interpretations of genetic variants [12,13,14].

In the era of PARP inhibitors such as olaparib and talazoparib to treat breast cancer, timely diagnosis of gBRCA mutations has become much more important. However, tests of gBRCA mutations by Myriad are more likely to be slower than those conducted by local laboratories because BRACAnalysis^®^ CDx can only be performed on specific serial number-controlled instruments at Myriad and must be analyzed at the Myriad facility in Utah [9]. It is not easy to replace Myriad’s BRACAnalysis^®^ CDx with local tests, because tests by individual laboratories lack reliability. To obtain timely diagnosis of gBRCA mutations, decrease costs associated with gBRCA mutation tests and successfully replace BRACAnalysis^®^ CDx with local tests, the validation of local laboratory tests is mandatory. If such validation is successful, these tests could be used as widely as the human epidermal growth factor receptor 2 (HER2) test. Here, we analyzed data indicating gBRCA mutation status in a sample of 31 patients based on tests conducted by a single local laboratory and by Myriad Genetics.

## 2. Materials and Methods

### 2.1. Study Population

**Figure 1:**
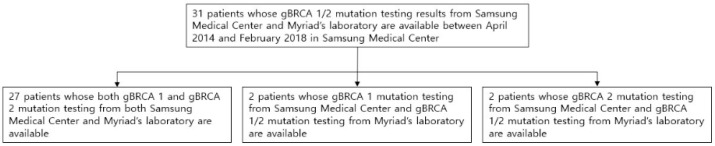
Study population.

Between April 2014 and February 2018, 31 patients (mean age, 41.8 years; range, 31–66 years) who underwent *BRCA 1/2* gene mutation testing by both Myriad and the Samsung Medical Center (Seoul, Korea) were enrolled in the present study. The pathology of breast cancers was assessed by the Samsung Medical Center. All samples used for *BRCA 1/2* gene mutation testing were whole blood. The samples were analyzed and interpreted by both Myriad and the Samsung Medical Center. Among them, we had full mutation test data for *BRCA 1/2* genes from Myriad and the Samsung Medical Center for 27 patients. We had full mutation test data for *BRCA 1/2* genes from Myriad but only *BRCA 1* gene mutation test data from the Samsung Medical Center laboratory for two patients. The remaining two patients had full mutation test data for *BRCA 1/2* genes from Myriad, but only *BRCA 2* gene mutation data from the Samsung Medical Center laboratory (Figure 1.). The latter four patients underwent either *BRCA 1* or *BRCA 2* gene mutation tests, respectively, for familial mutation studies.

### 2.2. Data Collection

The variables collected in this study were the following: age, sex, hormone receptor status of breast cancer tissue, HER2 and silver in situ hybridization (SISH) status, *BRCA 1/2* gene mutation test result from Myriad and the Samsung Medical Center.

### 2.3. Pathology

Breast tissue samples were embedded in paraffin and fixed in a 10% buffered formalin solution for 24–48 h at room temperature. Each sample was cut into 5-mm thick sections. Immunohistochemistry (IHC) staining for ER (1:200, 6F11, Novocastra Laboratories Ltd., Newcastle upon Tyne, UK), PR (1:100, clone, Novocastra Laboratories Ltd., Newcastle upon Tyne, UK), and HER2 (prediluted clone 4B5, Ventana Medical Systems, Inc., Tucson, AZ, USA) were performed. Slides were incubated with primary antibody at room temperature for 15 min (ER and PR), or at 37 °C for 32 min (HER2), respectively. Then, the Bond Polymer Refine Detection Kit (Leica Biosystems, Newcastle upon Tyne, UK, for ER and PR) or ultraView Universal DAB Detection Kit (Ventana Medical Systems, Inc. Tucson, AZ, USA, for HER2) were used for detection. A light microscope with ×100–200 magnification was used to evaluate stained slides. ER-positivity and PR-positivity were determined using a cut-off value of ≥1% stained tumor nuclei. HER2-positivity was determined if samples showed IHC 3+ positive staining or were SISH-positive. HER2 IHC 3+ was identified when there was a diffuse intense circumferential membrane “chicken-wire” staining pattern in >10% of the tumor. HER2 IHC 2+ was identified when there were tumors with circumferential membrane staining showing a thin pattern of staining and/or heterogeneity in a staining distribution of ≤10% of tumor cells. HER2 IHC 0 or 1+ was identified when tumors showed absent or weak membrane staining [15]. If samples exhibited IHC 2+ staining, HER2 SISH was used to evaluate HER2 gene status. HER2 SISH positivity was identified if the mean average HER2/CEP17 ratio was ≥ 2.0 or the average HER2 copy number was ≥ 6.0 signals/cell.

### 2.4. BRACAnalysis^®^ CDx

BRACAnalysis^®^ CDx requires samples of whole blood that are analyzed by PCR and Sanger sequencing for the detection of single nucleotide variants and small insertions and deletions (indels). In addition, multiplex quantitative PCR was used to detect large deletions and duplications. For *BRCA 1*, full sequencing of approximately 5400 base pairs comprising 22 coding exons and approximately 750 adjacent base pairs in the noncoding intervening (intron) sequences were performed. For *BRCA 2*, full sequencing of approximately 10,200 base pairs comprising 26 coding exons and approximately 900 adjacent base pairs in the noncoding intervening sequences were performed. Multiplex quantitative PCR was performed to detect large genomic rearrangements, including deletions and duplications, across all coding exons of *BRCA 1/2* genes, limited flanking intron regions and the proximal promoters [9].

Variants were identified as belonging to one of five categories: deleterious (pathogenic), suspected deleterious (likely pathogenic), variant of uncertain significance (VUS), favor polymorphism (likely not pathogenic), and polymorphism (not pathogenic). Another category, “unable to analyze”, described samples with insufficient quality or quantity of DNA.

Myriad uses an in-house variant classification program that was developed through reviews of new variants by a classification committee consisting of many experts in a variety of fields. This committee also reviewed new information for existing variants, following the American College of Medical Genetics and Genomics (ACMG) standards/guidelines [16]: identification of homozygous and compound heterozygous individuals, mutation co-occurrence, segregation, history-weighting algorithm, evolutionary conservation, functional or mRNA splice-site assays and population frequency [17].

### 2.5. BRCA 1/2 Gene Mutation Tests from Samsung Medical Center

At the Samsung Medical Center, the gBRCA 1/2 mutation test was implemented with full direct sequencing (PCR and Sanger sequencing). Multiplex ligation-dependent probe amplification (MLPA) to detect large rearrangements like large deletions and duplications was not routinely performed. MLPA was included if it was requested or large rearrangements were suspected, as when there were significant homozygous polymorphisms. However, full sequencing of *BRCA 1/2* genes was performed, as in Myriad’s BRACAnalysis^®^ CDx.

Variants were reported as one of three categories according to ACMG standards/guidelines, which are based on population data, computational and predictive data, functional data, segregation data, de novo data, allelic data, and other data [16]. However, ACMG standards/guidelines allow a large area of VUS. Thus, after categorization as VUS according to ACMG standards/guidelines, we recategorized variants according to the IARC working group multifactorial probability model, which is based on prior probability according to the Align-GVHD (Grantham variation/Grantham deviation) algorithm, segregation, co-occurrence in “trans”, personal and family history and histopathology [18]. For VUS, only variants of IARC classes 3, 4, 5 are reported, while variants of IARC class 1 and 2 are not. We referred to the Breast Cancer Information Core and GeneReviews^®^ [19,20].

### 2.6. Comparison of Variant Classifications

**Figure 2:**
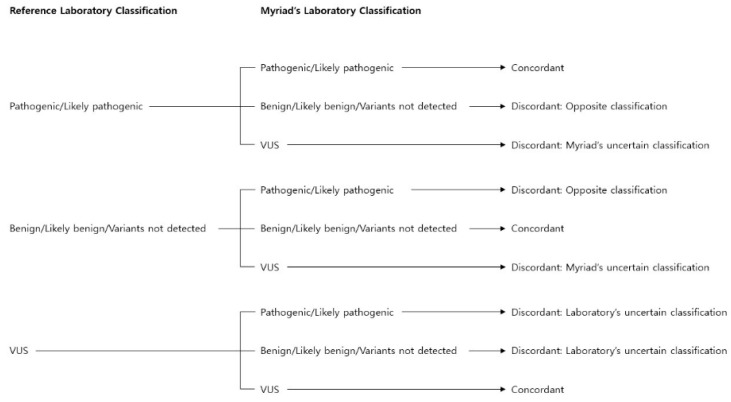
Comparison of variant classifications by the two laboratories. Reference: Gradishar W, Johnson K, Brown K, Mundt E, Manley S. Clinical Variant Classification: A Comparison of Public Databases and a Commercial Testing Laboratory. Oncologist 2017;22:797-803. Modified from Figure 2. Permitted by Journal The Oncologist.

Comparisons of variant classifications between Samsung Medical Center and Myriad were performed for the *BRCA 1* and *BRCA 2* genes following Gradishar W et al. [14] (Figure 2.). If “pathogenic variant/likely pathogenic variant” existed in a reference laboratory, only “pathogenic variant/likely pathogenic variant” from Myriad was defined as “Concordant”, while “benign variant/likely benign variant/variants not detected” by Myriad was defined as “Discordant: Opposite classification” and VUS from Myriad was defined as “Discordant: Myriad’s uncertain classification”. If “benign variant/likely benign variant/variants not detected” existed in a reference laboratory, only “benign variant/likely benign variant/variants not detected” by Myriad was defined as “Concordant”, while “pathogenic variant/likely pathogenic variant” from Myriad was defined as “Discordant: Opposite classification” and VUS from Myriad was defined as “Discordant: Myriad’s uncertain classification”. If a VUS existed in a reference laboratory, only the VUS from Myriad was defined as “Concordant”, while “pathogenic variant/likely pathogenic variant or benign variant/likely benign variant/variants not detected” by Myriad was defined as “Discordant: Laboratory’s uncertain classification”.

### 2.7. Statistical Analysis

Statistical analyses were performed using SPSS (version 18; IBM Corp., Armonk, NY, USA). Data are presented as mean ± standard deviation.

### 2.8. Ethical Considerations

The present study was approved by the Institutional Review Board of the Samsung Medical Center (approval no. 2019-02-064-002, 28 March 2019). The requirement for informed consent was waived due to the retrospective nature of the study.

## 3. Results

### 3.1. Patient Characteristics

The characteristics of 31 patients included in the study sample are presented in Table 1. The mean age was 41.8 years. All of the patients were female. Among 31 breast cancer tissue samples, estrogen receptor (ER), progesterone receptor (PR), and HER2 was positive in 14 (45.2%), 13 (41.9%), and one (3.2%) sample, respectively. Only one showed IHC 3+ HER2-positive staining. Among four (14.8%) with IHC 2+ HER2-positive staining, all HER2 SISH results were negative. Regarding subtypes of breast cancer, 17 (54.8%) had triple-negative breast cancer and 14 (45.2%) had ER-positive breast cancer.

### 3.2. gBRCA 1/2 Mutations

Among 31 patients enrolled, four had been tested for both BRCA 1 gene and BRCA 2 mutations by Myriad. Of these four patients, two were tested only for BRCA 1 mutations by the reference laboratory, while the other two were tested only for BRCA 2 mutations by the reference laboratory. The other 27 patients were tested for both BRCA 1 and BRCA 2 mutations by both Myriad and the reference laboratory.

BRCA 1 gene testing results for the 27 patients from Myriad and the reference laboratory are shown in Table 2. Among these 27 patients, 13 (48.1%) showed deleterious mutations of the BRCA 1 gene by Myriad, while the other 14 patients (51.9%) had no deleterious mutations. A deleterious large genomic rearrangement of the BRCA 1 gene was found in one patient (3.7%), 25 patients (92.6%) had no deleterious mutations and one (3.7%) patient’s sample was inadequate and could not be analyzed. Among these 27 patients, the reference laboratory obtained BRCA 1 gene sequencing results identical to Myriad’s. Deleterious mutations of the BRCA 1 gene were found in 13 patients (48.1%).

The test results of BRCA 2 gene of 27 patients by Myriad and the reference laboratory are shown in Table 3. Myriad detected deleterious mutations of the BRCA 2 gene in 10 (37.0%) patients and VUS in one (3.7%), while the other 16 patients (59.3%) had no deleterious mutations. There were 26 patients (96.3%) with no deleterious large genomic rearrangements of the BRCA 2 gene, while one (3.7%) patient sample was inadequate for analysis (see above). Among the 27 patients for whom the reference laboratory obtained results, the pathogenic or likely pathogenic variants identified in 10 patients (37.0%) were the same as those identified by Myriad. However, seven patients (25. 9%) were identified as VUS by the reference laboratory and the other 10 patients (37.0%) as having no deleterious mutations, conflicting with the results returned by Myriad.

### 3.3. Concordant Rate

**Figure 3:**
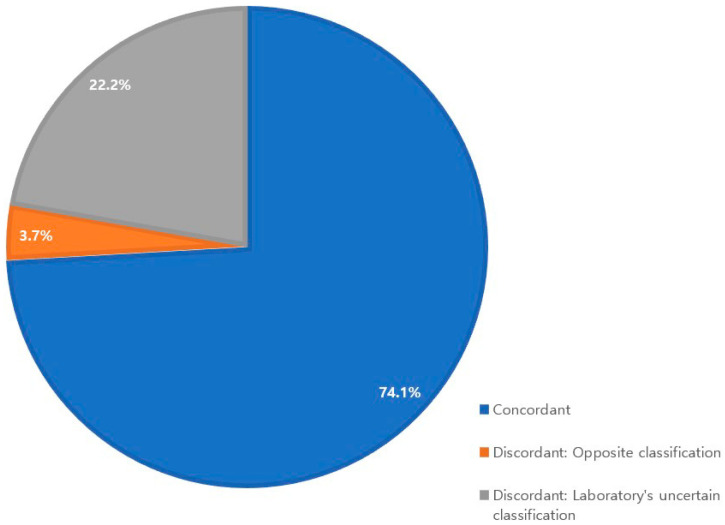
Concordant rates for 27 patients.

Concordant rates are shown in Figure 3. The concordant rate was 74.1% for 27 patients for whom gene mutation testing results for both BRCA 1 and BRCA 2 genes were available from the reference laboratory and Myriad.

“Discordant: Opposite classification” was obtained for just one patient (3.7%). This discrepancy was caused by the detection of a deleterious large genomic rearrangement of the BRCA 1 gene by Myriad, while the reference laboratory identified no deleterious BRCA 1 gene mutation through BRCA 1 gene sequencing and did not perform MLPA to find large genomic rearrangements.

Six patients (22.2%) were classified as “Discordant: Laboratory’s uncertain classification.” All of these discrepancies were due to the results of BRCA 2 gene sequencing.

Two patients were tested only for BRCA 1 gene mutations by the reference laboratory and for BRCA 1/2 gene mutations by Myriad. These two patients were concordant in deleterious mutations of BRCA 1. The other two patients were tested only for BRCA 2 gene mutations by the reference laboratory and for BRCA 1/2 gene mutations by Myriad. These two patients were also concordant, in deleterious mutations of the BRCA 2 gene. These four patients were tested only for either BRCA 1 or BRCA 2 gene mutations (but not both) by the reference laboratory because they had family histories of breast cancer and had already undergone mutation testing for familial mutation studies.

## 4. Discussion

In this study, we compared gBRCA 1/2 mutation test results obtained by a local reference laboratory and by Myriad, which offers a commercial test that is commonly used all over the world. “Discordant: Opposite classification” was found in only 3.7% of patients (just one case) since MLPA was not routinely performed by the reference laboratory. Our findings, which support the ability of local laboratories to conduct mutation tests, could contribute to the more widespread use of gBRCA 1/2 mutation testing. Such testing would help researchers to determine the regional prevalence of gBRCA 1/2 mutations, provide easier accessibility to mutation testing for people who are at risk of being gBRCA 1/2 mutation carriers and eventually treat breast cancer patients who have gBRCA 1/2 mutations with PARP inhibitors.

Because MLPA is time-consuming and expensive, it is not routinely performed by the reference laboratory and is applied only when there is a possibility of large rearrangements such as large deletions or duplications [21]. In addition, because large rearrangements are less likely in East Asians compared to other populations (nonAshkenazi Jewish or Latin American/Caribbean), MLPA is not routinely performed [22,23,24]. However, even though MLPA tests are performed for high-risk patients by the reference laboratory, there was one missed patient. The present study showed the importance of performing MLPA routinely with *BRCA* sequencing. MLPA was not performed in one patient for either *BRCA 1/2* gene by Myriad because of an inadequate blood sample. This indicates the need for adequate sample collection to routinely test for large rearrangements of genes.

Six patients (22.2%) were classified as “Discordant: Laboratory’s uncertain classification”. Among them, three had results of VUS from the reference laboratory but no mutations detected by Myriad’s laboratory. The other three patients had results of VUS from the reference laboratory but results of “favor polymorphism” from Myriad. All six discrepancies were due to the results of *BRCA 2* gene sequencing. Despite the identical sequences of the *BRCA 2* gene obtained by the laboratories, the interpretations of the results were different. This might be due to differences in libraries, references and classification methods between laboratories. Standards for the genetic testing of *BRCA 1/2* genes do not exist, including methods, types of software and analytical tools, criteria for interpreting test results, databases utilized, categories, types of clinical information referenced or types of references used for considering population frequency [25].

The mean time required to obtain results from the reference laboratory was longer than that from Myriad, 42.0 days vs. 10.5 days, respectively, even though samples were sent to Myriad, located in the United States, from Korea. This might be because there were not enough samples for the reference laboratory to run at once, compared to Myriad’s laboratory, which analyzes samples from all over the world. If the number of samples analyzed by local laboratories increases, local analysis will become more time and cost-efficient. To achieve this goal, local genetic testing must be approved after further evaluation and validation.

The major limitation of genetic testing by the reference laboratory was the rate of VUS. All six VUS cases identified by the reference laboratory were defined more precisely as “no mutation” or “favor polymorphism” by Myriad’s laboratory. However, this does not mean that the reference laboratory did not provide accurate results. The Food and Drug Administration of the United States maintains that novel mutations are still being identified and classified, and that deleterious mutations can change and be reclassified with time, and there are many studies showing conflicting interpretations of genetic variants [9,12,13,14]. An example is *BRCA 1* c.5339T>C (L1780P), for which recent studies support the reclassification of *BRCA 1* c.5339T>C (L1780P) variants as “likely pathogenic” [26,27]. In addition, interpretation of VUS may differ according to the classification system [28]. Even though it is not clear which laboratory is more accurate, VUS results would burden both physicians and patients by increasing anxiety and requiring further expense for additional genetic testing, follow-up or unnecessary procedures such as preventive operations [29]. This indicates that further research and standardization of genetic testing for the *BRCA 1/2* gene are needed.

The largest limitation of this study is that it was not a high-level prospective comparison study. It was retrospectively designed and so is missing some data. Despite this, we evaluated the feasibility of using local laboratory genetic diagnostics of the *BRCA* mutation. Further well-designed prospective studies with a more sufficient number of samples should assess the possibility of expansion of genetic diagnostics of the gBRCA mutation.

## 5. Conclusions

In this study, we compared the results of gBRCA 1/2 mutation testing from a local reference laboratory and Myriad and found that the “Discordant: Opposite classification” was only 3.7%. However, considering that “Discordant: Laboratory’s uncertain classification” was 22.2%, further research and standardization of genetic testing for *BRCA 1/2* genes are required. If validation of local testing for *BRCA 1/2* gene mutations is successful, more widespread use of such testing will contribute to knowledge about regional prevalence, eventually facilitate the screening of patients who may be gBRCA 1/2 mutation carriers and allow application of proper treatments for breast cancer.

## Figures and Tables

**Table 1:** Characteristics of the 31 patients enrolled in this study.

Variable	Value
Mean age (range), years	41.8 (31–66)
Sex, *n* (%)	
Male	0 (0.0%)
Female	31 (100.0%)
Breast cancer hormone receptor, *n* (%)	
Estrogen receptor (ER)	14 (45.2%)
Progesterone receptor (PR)	13 (41.9%)
Human epidermal growth factor receptor 2 (HER2)	1 (3.2%)
Subtype, *n* (%)	
Triple-negative	17 (54.8%)
ER-positive	14 (45.2%)
HER2-positive	0 (0.0%)

**Table 2:** Germline BRCA 1 mutation test results from Myriad and Samsung Medical Center.

Variable	Value
*BRCA 1* gene sequencing from Myriad’s laboratory, *n* (%)	
Pathogenic/likely pathogenic variant	13 (48.1%)
VUS	0 (0.0%)
Benign/likely benign/variants not detected	14 (51.9%)
*BRCA 1* gene large genomic rearrangement from Myriad’s	
laboratory, *n* (%)	
Pathogenic/likely pathogenic variant	1 (3.7%)
Benign/likely benign/variants not detected	25 (92.6%)
Unable to analyze	1 (3.7%)
*BRCA 1* gene sequencing from Samsung Medical Center, *n* (%)	
Pathogenic/likely pathogenic variant	13 (48.1%)
VUS	0 (0.0%)
Benign/likely benign/variants not detected	14 (51.9%)

Abbreviation: VUS, variant of uncertain significance.

**Table 3:** Germline BRCA 2 mutation test results from Myriad and Samsung Medical Center.

Variable	Value
*BRCA 2* gene sequencing from Myriad’s laboratory, *n* (%)	
Pathogenic/likely pathogenic variant	10 (37.0%)
VUS	1 (3.7%)
Benign/likely benign/variants not detected	16 (59.3%)
*BRCA 2* gene large genomic rearrangement from Myriad’s	
laboratory, *n* (%)	
Pathogenic/likely pathogenic variant	0 (0.0%)
Benign/likely benign/variants not detected	26 (96.3%)
Unable to analyze	1 (3.7%)
*BRCA 2* gene sequencing from Samsung Medical Center, *n* (%)	
Pathogenic/likely pathogenic variant	10 (37.0%)
VUS	7 (25.9%)
Benign/likely benign/variants not detected	10 (37.0%)

Abbreviation: VUS, variant of uncertain significance.

## Data Availability

Data available on request due to restrictions of privacy.
